# The impact of mobile health interventions on service users’ health outcomes and the role of health professions: a systematic review of systematic reviews—protocol

**DOI:** 10.1186/s13643-024-02624-y

**Published:** 2024-07-27

**Authors:** Fathiya Alkhuzaimi, Deborah Rainey, Christine Brown Wilson, Jacqueline Bloomfield

**Affiliations:** 1 School of Nursing and Midwifery Queen’s University Belfast, University Rd., Belfast, BT7 1NN Northern Ireland; 2 School of Nursing and Midwifery, The University of Sydney, Susan Wakil Health Building Western Avenue Camperdown, New South Wales, Australia

**Keywords:** Mobile health, Digital health, Patients’ outcomes, Long-term conditions, Health professions

## Abstract

**Background:**

Mobile health tools have gained prominence in global health care in recent years. Mobile health (mHealth) interventions have demonstrated their impact on managing healthcare service users’ health. A pilot search revealed many systematic reviews on the effectiveness of mobile health tools on service users’ health outcomes. However, how the role of healthcare professionals in promoting the adoption of mobile health may lead to improved outcomes needs to be clarified. Therefore, this systematic review aims to synthesise existing systematic reviews that examine both the impact of mobile health interventions on service users’ outcomes and the role of healthcare professionals in facilitating the adoption of mobile health solutions.

**Methods:**

Five electronic databases will be searched: EMBASE, CINHAL Plus, MEDLINE, Web of Science, and the Cochrane Library for systematic reviews exploring the impact of mobile health interventions on service users’ outcomes and the role of healthcare professionals in facilitating the adoption of mobile health solutions. Systematic reviews published in English dated from January 2015 to June 2024 will be included. Screening and selection of the reviews against inclusion and exclusion criteria will be performed by three independent reviewers, as well as data extraction and quality assessment.

**Discussion:**

Current systematic reviews in mHealth have primarily focused on assessing the effectiveness of mHealth interventions for managing a range of conditions. While these reviews provide valuable input into the outcomes for mHealth, more is needed to know about the impact of the involvement of health professions on service users’ outcomes when adopting mHealth. This systematic review of systematic reviews aims to bridge this critical gap in the literature by critically appraising and synthesising the evidence of mHealth interventions’ impact on service user outcomes and the level of involvement of health professionals.

**Systematic review registration:**

PROSPERO CRD 42023414435.

## Background

The exponential growth of chronic diseases and the ageing population worldwide pose increasing challenges to adequate healthcare provision [1]. The crisis of the COVID-19 pandemic has highlighted the practical impact of digital technologies to provide health solutions [2]. Various digital technologies are being developed and used to help in the medical field, including mobile health technology, which has been harnessed in healthcare services [3]. Mobile health (mHealth)involves delivering comprehensive medical and health aid to individuals seeking healthcare via mobile phones, laptops, tablets, and wearable devices [4]. Mobile health technology significantly influences individuals’ health-related behaviours, including physical activity, dietary choices, alcohol consumption, sexual conduct, and adherence to medication regimens [5]. There has been significant growth in the body of literature concerning mHealth over the last decade [6], with the USA and the UK being the most active countries in mHealth research [7]. mHealth interventions are considered powerful tools that have led to revolutionary changes in digital health, particularly in access, monitoring, education, and intervention [8]. For example, a recent systematic review found that mHealth interventions could monitor patients’ conditions remotely, deliver clinical consultation, enhance their engagement, and increase their autonomy in their health management [9].

Furthermore, there is considerable potential for mHealth in various healthcare domains encompassing preventive measures and wellness initiatives, remote and self-diagnostic capabilities, monitoring medication adherence, dissemination of health-related information, and managing chronic diseases [10]. Therefore, mHealth interventions have received recognition and support from global regulatory institutions [11]. For example, the World Health Organisation (WHO) and the National Institute for Health and Care Excellence (NICE) have both acknowledged the potential benefits of mHealth on patient outcomes, disease prevention, and reducing the workload of healthcare providers [12]. The European Commission also described using mHealth as crucial to addressing the healthcare system’s challenges in Europe [13]. In the United Kingdom (UK), National Health Services (NHS) Digital and other organisations have highlighted the importance of mHealth interventions in managing health problems to meet the high public demand for health services [14].

During the outbreak of COVID-19, mHealth significantly impacted the management of health issues. In India, patients’ engagement and utilisation of mHealth increased dramatically during the lockdown [15]. In Brazil, a randomised controlled trial (RCT) found that using mHealth tools to effectively communicate with the public enhanced people’s adherence to preventive measures for COVID-19 [16]. Similarly, in Australia, mHealth interventions were used to provide information about symptoms of COVID-19, prevention, vaccination, and changing behaviour with lifestyle modification for older people [17]. Likewise, other studies worldwide have reported that mHealth technology successfully fought the COVID-19 pandemic [18].

Furthermore, in the UK, the NHS long-term plan (2019–2024) recommends that health professionals be supported to develop digital literacy to use mobile access to digital services. [19]. The latest evidence indicates that integration is crucial, as is assuring the ethical implementation of these technologies [20]. The subsequent steps entail a synthesised methodology that combines innovative elements.

However, the global literature from high-income countries suggests that there is hesitation among health professionals to implement and advocate for the use of digital health technologies in their practice [21–23]. This was echoed by a study conducted in Catalonia, which revealed that only 6.5% of the surveyed nurses consistently advocated for integrating digital technology into their regular provision of patient care [24]. Because there is a breadth of literature in digital health, especially in mobile health, this review aims to conduct a systematic review of systematic reviews to assess the current state of evidence on the impact of mHealth adoption on service users’ outcomes and the influence of health professions in the adoption of mHealth on their service users.

### Review questions

1. What is the state of the systematic review evidence on interventions designed to influence service user adoption of mobile health to improve health outcomes?
2. What is the state of the systematic review evidence on interventions that are actively attempting to engage health professions to improve service user outcomes with mobile health?
3. Which patient health outcomes are addressed in those reviews of evidence attempting to engage/health professionals when using mobile health?
4. What is the methodological quality of the systematic reviews of evidence of interventions attempting to engage health professionals in using mobile health technology that is explicitly designed to improve service user health outcomes?

#### Search strategy

An initial scoping search was undertaken to develop the search terms for this review (Table 1). The initial search will combine the search terms across multiple databases. Five electronic databases will be used: EMBASE, CINHAL Plus, MEDLINE, Web of Science, and the Cochrane Library. For a systematic review of systematic reviews, The Joanna Briggs Institute (JBI) recommends searching for research syntheses between 5 and 10 years that will reveal original research from 30 + years ago [25]. Furthermore, da Silva and colleagues undertook a state-of-science review on health in 2015 [26], with further research underpinning the European Green paper [27]. Therefore, the literature search was limited to publications from 2015 to 2023 to identify the most up-to-date and comprehensive breadth of systematic reviews. A revised search will be carried out from January 2023 until June 8, 2024, to incorporate the latest studies.

**Table 1:** Combination of search terms using Boolean logic

“Health care professional*” OR “Health care provider*” OR “health Professional*” OR “Health Personnel (MeSH)”OR “caregiver*””OR “ “Health profession*”	“Digital Health” OR “mHealth” OR “Telemedicine(MeSH)” OR “Telehealth(MeSH)” OR “Mobile Health” “ Informatics(MeSH)” OR “digital application*”	“Patient Care(MeSH)” OR “patient outcome*” OR “patient centred care”	“Systematic Review(MeSH)”

#### Study designs

Systematic reviews published in peer-reviewed English language Journals will be included.

#### Identification of search terms

The following four search terms will be used in this review: health care professional*, digital health, patient care, and systematic review augmented by MESH terms and combined using Boolean logic (Table 1).

#### Inclusion and exclusion criteria

Studies will be selected based on the following criteria and a list of inclusion and exclusion criteria, as illustrated in Table 2.

**Table 2:** The inclusion and exclusion criteria of this study

Inclusion	Exclusion
Interventions involving mobile health with service users	Interventions involving telehealth; telemedicine, clinical decision-making tools, communication tools between health care providers; and digital care delivery
Mobile Health interventions measuring service user outcomes	Non-intervention studies
Engagement of health profession using mobile health to improve service user outcomes	
Published from 2015 to 2024	Studies concerning mobile health but not focus on patient outcomes
Published in the English language	Reviews not following a systematic search strategy
Systematic reviews	Single research studies

#### Population

This systematic review examines two primary populations: healthcare service users who utilise mobile health (mHealth) tools for managing their health outcomes and healthcare professionals who advocate for using mHealth tools among their service users to enhance health outcomes. This multifaceted focus enables a thorough evaluation of the effects of mHealth interventions on both service users and the involvement of healthcare professionals in promoting the adoption of mHealth. This review excludes studies that focus only on telemedicine or consider mHealth tools only as communication aids among healthcare professionals, as they fall beyond the defined scope of this review.

#### Intervention

The review will focus on interventions involving mHealth with service users, mHealth interventions measuring service user outcomes, and the engagement of health professionals using mobile health to improve service user outcomes. Systematic reviews not focused on mHealth interventions or service user outcomes will be excluded.

#### Comparator

This review will focus on mHealth interventions compared with usual care. Usual care can be delivered with non-mobile health technology.

#### Primary outcomes

The main outcome of this review has two primary outcomes:

1. To assess the impact of mobile health interventions on health-related outcomes of service users. Examples of relevant outcomes may include enhancing service user self-management of wellness and disease prevention and improving their accessibility to health services anxiety, adherence to medications, hospital admission, follow-up, behaviour change, and improving their accessibility to health services.
2. How the role of health professions in promoting the adoption of mobile health influences service users’ health-related outcomes. Examples may include adoption and utilisation rates, service users’ satisfaction and engagement, health literacy and empowerment of health professions, health outcomes for the service users, and the cost-effectiveness of promoting mobile health services by health professionals.

#### Additional outcomes

Factors affecting service users in adopting mobile health and health professions in promoting mobile health to their service users.

#### Data exclusion

The characteristics of excluded reviews and reasons for exclusion are listed in a table.

#### Screening and selection

**Fig. 1:**
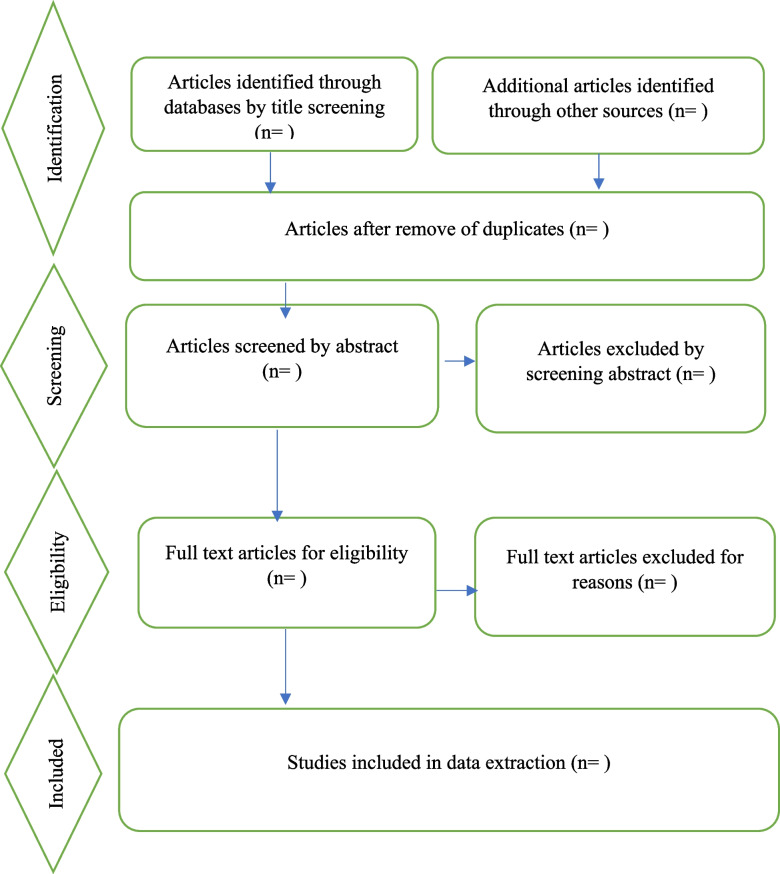
PRISMA chart

Records were collated, and duplicates were removed using Endnote software. The data will be managed using Microsoft Excel software. A team of four reviewers will review title abstracts and full papers: three reviewers will independently screen the titles, abstracts, and papers, with a third reviewer available for disagreements. The selection process for the papers will be recorded in detail in the PRISMA-P flow diagram [28] as indicated in Fig. 1.

#### Data extraction

This review will implement a rigorous data extraction procedure to systematically collect and categorise essential data from the included reviews. The purpose of this process is to ensure consistency and accuracy in the analysis. A standardised data extraction form will be developed by the reviewers to provide guidance for the reviewers. To ensure comprehensive and unbiased data extraction, four reviewers will extract the data independently. Interrater reliability will be assessed to ensure consistency. The data extraction process will require a thorough examination of each study included. The data collected from these studies will be organised into tables to enhance clarity and accessibility for further analysis. The following details will be extracted from each study: author names, publication year, journal name, type of systematic review (if applicable), country of origin, range of years covered by the included studies, study settings, quality appraisal tool used, number of included studies, types of health conditions investigated, types of mHealth tools used, intervention descriptions, measured outcomes, and relevant role of health professions. A well-organised and thorough data extraction process is crucial for ensuring transparency, rigour, and reliability in our systematic review. This process ultimately enhances the validity of our research findings.

#### Data quality assessment

Four reviewers will independently assess the quality of systematic reviews by using AMSTAR 2 (A MeaSurement Tool to Assess Systematic Reviews), and if there are any discrepancies, they will discuss them with the third reviewer. The AMSTAR 2 is a critical appraisal tool to assess the methodological quality of systematic reviews [29]. It is composed of 16 questions, including seven critical domains with an overall rating based on the weaknesses of critical domains. The results of the risk of bias assessment will be documented in the findings and discussion.

#### Data synthesis

A narrative review will be used to synthesise the findings of systematic reviews to address the review questions. This review will apply thematic analysis to identify main themes and subthemes emerging from the findings based on similarities and differences of mHealth interventions on service users’ outcomes, types of interventions, types of mHealth tools, types of outcomes, the impact of health professions on service users’ outcomes, factors affecting adoption of mHealth by the service users, and factors affecting health professions in promoting the adoption of mHealth among their service users. Thematic analysis will be carried out manually by four independent reviewers. A coding technique will be used to classify and code the identified themes and subthemes. The primary research questions and topics of focus will inform the creation of this framework. The coding process will be progressive, with code reviews occurring at various stages. Periodically throughout the analytic process, intercoder reliability will be evaluated to ensure rigour and consistency. Coding discrepancies will be discussed and resolved at regularly scheduled meetings. Existing themes from the reviews will be discussed with the support of evidence from systematic reviews. Comparisons and contrasts will be highlighted. These findings will be critically analysed, taking into consideration the strengths and limitations of the included reviews. The implications of these findings for clinical practice and the identification of any gaps in the existing reviews will be discussed. The synthesis of the findings will provide recommendations for clinical practice, policy, and future research.

## Discussion

Current systematic reviews in mHealth have primarily focused on assessing the effectiveness of mHealth interventions for managing disease conditions. While these reviews provide valuable input into the outcomes of such interventions, more information is needed about the impact of the involvement of health professions on service users’ outcomes when adopting mHealth. This systematic review of systematic reviews aims to bridge this critical gap in the literature by critically appraising and synthesising the evidence of mHealth interventions’ impact on service user outcomes and the level of involvement of health professionals. A recent systematic review and meta-analysis highlighted that mHealth supports health professionals in making clinical decisions, managing and communicating with patients, and monitoring patients remotely [30]. Despite the clear benefits highlighted by various studies, the involvement of health professionals in promoting mHealth to their patients is limited [31, 32]. There is a large body of evidence in the field of mHealth interventions in the field of digital health. Therefore, this review will review the current evidence on mHealth interventions on service user outcomes while providing a unique perspective on the health professions’ influence on mHealth adoption and service user outcomes. This review will contribute significantly to the existing body of knowledge by understanding the role of health professions in guiding health service users towards mHealth solutions, exploring the impact of the involvement of health professions on service users’ outcomes to inform clinical practice, and recommending future research. Additionally, findings from this systematic review of systematic reviews will inform policymakers on policy and framework considerations to enhance the adoption of mHealth interventions into practice**.**

## Data Availability

Not applicable.

## Abbreviations

AMSTAR2: A Measurement Tool to Assess Systematic Reviews
JBI: The Joanna Briggs Institute
CINAHL: Cumulative Index to Nursing and Allied Health Literature
MEDLINE: Medical Literature Analysis and Retrieval System Online
MeSH: Medical Subject Headings
PRISMA: Preferred Reporting Items for Systematic Reviews and Meta-Analyses
EMBASE: Excerpta Medica database
PROSPERO: International prospective register of systematic reviews
NICE: The National Institute for Health and Care Excellence
WHO: World Health Organisation
NHS: National Health Service
